# Naïve T Cells Re-Distribute to the Lungs of Selectin Ligand Deficient Mice

**DOI:** 10.1371/journal.pone.0010973

**Published:** 2010-06-04

**Authors:** John R. Harp, Thandi M. Onami

**Affiliations:** Department of Microbiology, University of Tennessee, Knoxville, Tennessee, United States of America; Hannover School of Medicine, Germany

## Abstract

**Background:**

Selectin mediated tethering represents one of the earliest steps in T cell extravasation into lymph nodes via high endothelial venules and is dependent on the biosynthesis of sialyl Lewis X (sLe^x^) ligands by several glycosyltransferases, including two fucosyltransferases, fucosyltransferase-IV and –VII. Selectin mediated binding also plays a key role in T cell entry to inflamed organs.

**Methodology/Principal Findings:**

To understand how loss of selectin ligands (sLe^x^) influences T cell migration to the lung, we examined fucosyltransferase-IV and –VII double knockout (FtDKO) mice. We discovered that FtDKO mice showed significant increases (∼5-fold) in numbers of naïve T cells in non-inflamed lung parenchyma with no evidence of induced bronchus-associated lymphoid tissue. In contrast, activated T cells were reduced in inflamed lungs of FtDKO mice following viral infection, consistent with the established role of selectin mediated T cell extravasation into inflamed lung. Adoptive transfer of T cells into FtDKO mice revealed impaired T cell entry to lymph nodes, but selective accumulation in non-lymphoid organs. Moreover, inhibition of T cell entry to the lymph nodes by blockade of L-selectin, or treatment of T cells with pertussis toxin to inhibit chemokine dependent G-coupled receptor signaling, also resulted in increased T cells in non-lymphoid organs. Conversely, inhibition of T cell egress from lymph nodes using FTY720 agonism of S1P1 impaired T cell migration into non-lymphoid organs.

**Conclusions/Significance:**

Taken together, our results suggest that impaired T cell entry into lymph nodes via high endothelial venules due to genetic deficiency of selectin ligands results in the selective re-distribution and accumulation of T cells in non-lymphoid organs, and correlates with their increased frequency in the blood. Re-distribution of T cells into organs could potentially play a role in the initiation of T cell mediated organ diseases.

## Introduction

Lymphocytes are highly migratory cells. Uncovering pathways that allow these cells to access tissues not only facilitates our understanding of cell migration, but deepens our understanding of the relationship between immunity and immunopathology. Blood borne leukocytes, such as T lymphocytes, are recruited to extra-vascular sites, and these processes are highly regulated so that distinct cell populations are delivered to intended site(s) in the proper physiological or pathological context (reviewed in [1], [2], [3]). Imperfections in these processes may lead to qualitatively or quantitatively inappropriate delivery of cells to tissue sites that may result in inflammatory pathological processes (reviewed in [3]). Several leukocyte adhesion deficiency (LAD) diseases in humans have been described, including LAD II, a congenital immunodeficiency disease characterized by patients having defective production of fucosylated ligands necessary for selectin mediated tethering [4], [5], [6], [7].

Selectins (E-selectin, P-selection, and L-selectin) are type I transmembrane glycan binding proteins (GBPs), or C-type lectins, that regulate leukocyte-endothelial cell adhesive interactions, critically involved in T lymphocyte trafficking from the blood to extra-vascular compartments [3]. Selectins interact selectively with sialylated fucosylated lactosamine structures (sialyl Lewis x or sLe^x^) of membrane-associated mucin type glycoproteins such as GlyCAM-1, CD34, and MAdCAM-1 via binding by amino terminal carbohydrate recognition domains (CRDs). The α1, 3 fucosyltransferases catalyze the synthesis of the sLe^x^ moiety, and thus these enzymes are essential in the elaboration of selectin ligands, although additional modification by sulfotransferases contributes to L-selectin binding [8], [9], [10], [11]. Naïve and central memory T lymphocytes express leukocyte selectin, or L-selectin, while activated T lymphocytes downregulate L-selectin, but begin to express E- and P- selectin ligands as a consequence of inducible expression of α1, 3 fucosyltransferases [12], [13], [14]. High endothelial cells which line functionally distinct high endothelial venules (HEVs) in different lymph nodes, constitutively express α1, 3 fucosyltransferases, and this allows shear-dependent adhesive interactions between lymphocytes and selectin ligands on HEVs [15], [16]. Following initial selectin-mediated tethering of T cells, chemokine receptor binding of chemokines, such as CCR7 binding CCL21, activates G-coupled receptor signaling, which induces a conformational change of T cell integrins, such as LFA-1 [17]. Binding of high affinity LFA-1 on T cells leads to firm arrest and facilitates transendothelial migration into the lymph node (LN) [17], [18].

Mice lacking the α1, 3 fucosyltransferases were generated several years ago and demonstrate loss of selectin binding activity, impaired T cell migration to draining lymph nodes but normal migration to spleen, as well as impaired T cell extravasation to skin in both contact hypersensitivity and viral models [12], [19]. However, knockout mice of one or both enzymes are able to mount potent immune responses as evidenced by clearance of lymphocytic choriomeningitis virus (LCMV), vesicular stomatitis virus (VSV), and vaccinia viral infection in brain and ovaries, despite reduction of lymph node T cell numbers [12], [19], [20]. Moreover, examination of fucosyltransferase-VII and-IV double knockout mice (FtDKO) for immunity following infection with *Mycobacterium tuberculosis* found that FtDKO mice had significantly diminished numbers of activated CD8 and CD4 T cells in the draining lymph node of the lung (mediastinal lymph node, MedLN), but had effective granuloma responses and similar recruitment of activated T cells into the lung at later stages of infection [21]. Importantly, *Mycobacterium tuberculosis* infection of FtDKO mice demonstrated that iBALT develops similarly in the lungs of FtDKO mice, and express CXCL13, CCL21, and CCL19 chemokines [21]. This reduction in T cells in the MedLN of FtDKO mice, even under conditions of chronic inflammation by an intracellular pathogen, convincingly suggests that no major alternative mechanism of cell recruitment compensates for the deficiency of α1, 3 fucosyltransferases in T cell homing to the LN [21].

We were interested in determining how loss of selectin ligands affects immunity at mucosal sites [22]. Here we report our discovery that FtDKO mice display a significant increase in naïve T cell populations located in the lung. This phenotype has never been reported in these mice, so we further explored the contribution of selectin ligands to T cell migration into non-lymphoid organs. We examined T cell trafficking in FtDKO mice under non-inflammatory conditions, as well as following inflammation due to viral infection. The data presented here suggests that in FtDKO mice, T cells selectively accumulate in non-lymphoid organs under non-inflammatory conditions. However, under inflammatory conditions, T cell trafficking of Ag-specific effector and memory T cell populations to non-lymphoid organs was reduced in FtDKO mice, consistent with previous studies [23], [24], [25]. Finally, to investigate why naïve T cells would preferentially accumulate in non-lymphoid organs of FtDKO mice under non-inflammatory conditions, we considered two explanations—1) a specific increase in trafficking due to loss of selectin ligand expression in the lungs of FtDKO, making them more permissive to T cell entry, or 2) a non-specific increase in trafficking to non-lymphoid organs due to T cell re-distribution when cells cannot enter LNs. We tested the latter hypothesis and our results are consistent with this hypothesis. Treatments that block T cell entry into the LN result in selectively increased numbers of T cells in non-lymphoid organs. Conversely, treatments that blocked T cell exit from the LN showed reduced T cell trafficking to non-lymphoid organs. Thus, under conditions where T cell entry to LNs is impaired, T cells may accumulate in the perivascular or interstitial compartments of the lung, and this altered distribution could possibly play a role in the initiation of lung diseases.

## Results

### Increased numbers of naïve T cells in the lungs of FtDKO mice

**Figure 1:**
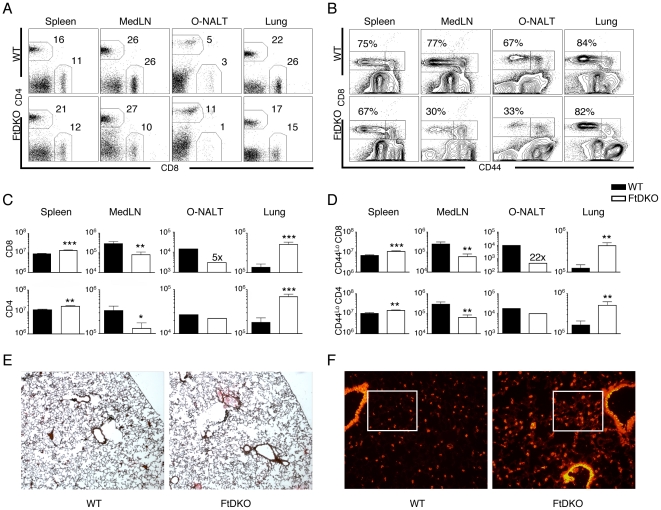
Increased numbers of naïve T cells in lungs of FtDKO mice. Lymphocytes were isolated from WT and FtDKO mice. *A* and *B*, Representative FACS analysis of organs from WT or FtDKO mice. Cells were stained with CD4, CD8 and CD44 mAbs. Numbers indicate percent of CD4, CD8, or CD44^Lo^ CD8 T cells. *C*, Enumeration of CD8 and CD4 T cells in indicated organs. *D*, Enumeration of CD44 ^Lo^ CD8 or CD44 ^Lo^ CD4 T cells in indicated organs. *E*, Photomicrographs of representative H & E staining of WT and FtDKO lungs at ×40 magnification. F. Photomicrographs of representative immuno-histology staining of T cells using Thy1 of WT and FtDKO lungs at ×200 magnification. Data was collected from n = 6 WT and n = 7 FtDKO in two independent experiments. For O-NALT, one experiment is shown where cells were pooled from n = 4 mice/group. The unpaired Student *t* test was used to compare groups. (*p<0.05, ** p<0.01, and *** p<0.001).

We isolated lymphocytes from organs of naïve WT and FtDKO mice and analyzed CD4 and CD8 T populations at each site. As expected, CD4 and CD8 T cell populations were significantly reduced (∼70%) in mediastinal lymph node (MedLN) (Figure 1A, C). CD8 T cell populations were also reduced in the organized nasal associated lymphoid tissue (O-NALT). Surprisingly, we observed ∼5-fold increase in T cell numbers in the lungs of FtDKO mice, and we also consistently observed a modest increase in T cell numbers in the spleen (∼20% increase) (Figure 1A, C). B cell populations were also increased in the lung, about 2-fold (data not shown). Phenotypic analysis of T cell populations in organs of the FtDKO mice reveal that similar to WT mice, the majority of T cells in the spleen and lung were naïve (CD44^lo^ and CD62L^high^) (Figure 1B and data not shown). However, in the MedLN and O-NALT of FtDKO mice, there was a significant decrease in naïve (CD44^lo^) T cell populations (Figure 1B, D).

Since we saw increased numbers of T cells in the lungs of FtDKO mice, we hypothesized that similar to CCR7-/- mice, FtDKO mice may show evidence of neo-lymphoid follicles or induced bronchus-associated lymphoid tissue (iBALT) in the lungs [26]. However, H&E analysis of lung sections from FtDKO showed no evidence of iBALT in any lung sections examined (Figure 1E). Analysis of T cells in lung sections suggested increased T cells in the perivascular compartment, consistent with the observed increase in T cell numbers by FACS analysis (Figure 1F). Taken together, these observations raised the possibility that naïve T cells in FtDKO mice were re-distributing to other organs, including non-lymphoid organs such as the lung.

### Migration of Ag-specific CD8 T cells to inflamed lung is impaired in FtDKO mice

The role of selectin mediated recruitment of leukocytes to inflamed tissues is well established [23], [24], [27]. Following inflammatory insult, E- and P-selectin are upregulated on inflamed endothelial cells allowing for the recruitment of selectin ligand expressing leukocytes. Naïve T cells express L-selectin, but not selectin ligands, while activated T cells downregulate L-selectin, both transcriptionally and via proteolytic cleavage, and induce expression of α1, 3 fucosyltransferases, resulting in selectin ligand expression [14], [28], [29], [30].

**Figure 2:**
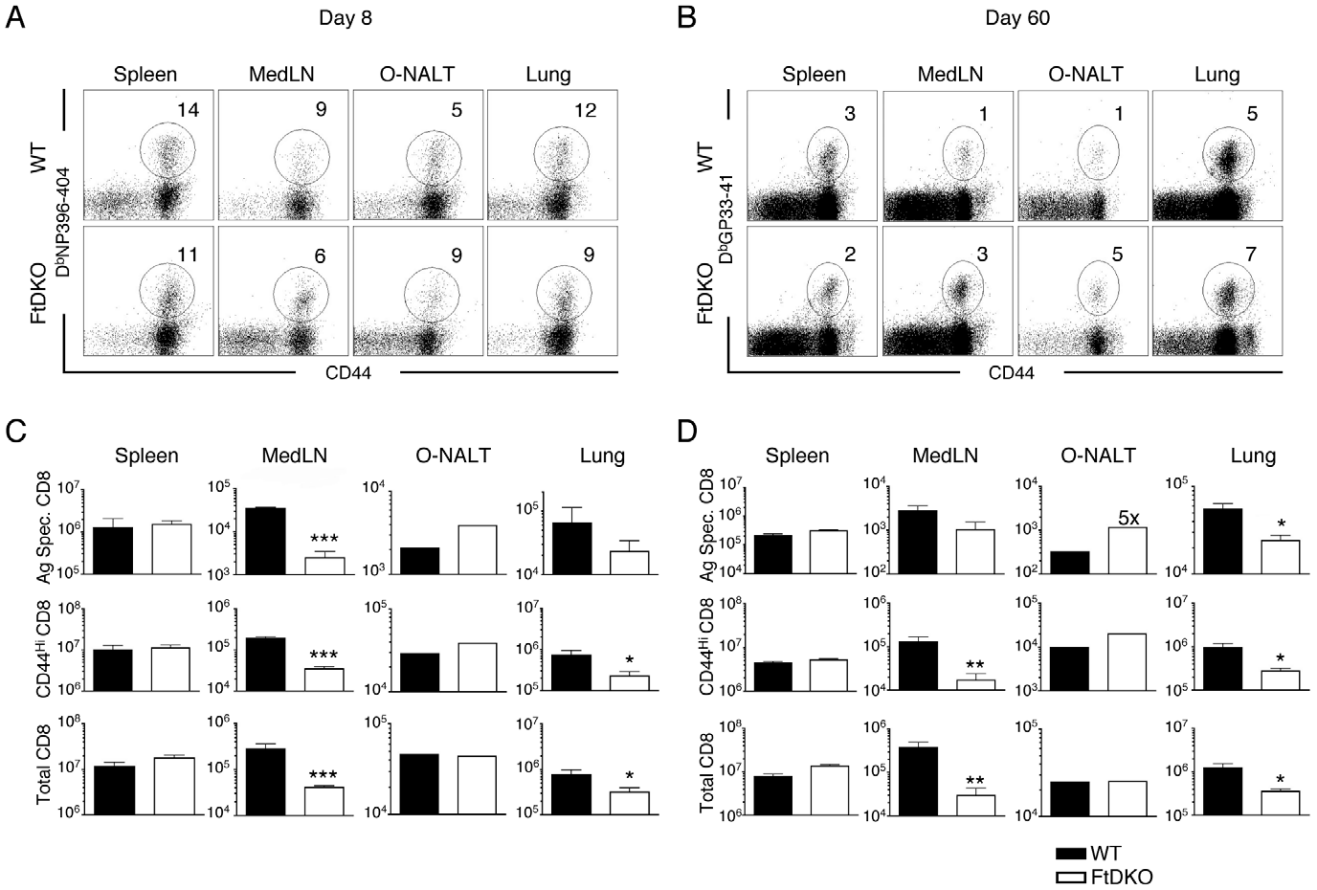
Ag-specific CD8 T cells are reduced in inflamed lung of FtDKO mice. WT or FtDKO mice were infected with LCMV-Armstrong i.p and lymphocytes were isolated on day 8 or day 60 p.i. *A*, Representative FACS analysis of single cell suspensions from indicated organs on day 8 p.i. Cells were stained with CD8, CD44 and D_b_NP396-404 tetramer. Gating on CD8 T cells, numbers in FACS panels indicate percent of Ag-specific CD8 T cells in organs. *B*, Representative FACS analysis of single cell suspensions from indicated organs on day 60 p.i. Cells were stained with CD8, CD44 and D_b_GP33-41 tetramer. *C*, Enumeration of Ag-specific, CD44^high^, and total CD8 T cells in WT or FtDKO mice at day 8. *D*, Enumeration of Ag-specific, CD44^high^, and total CD8 T cells in WT or FtDKO mice at day 60. For day 8, n = 2 WT and n = 3 FtDKO. For day 60, n = 4 WT and n = 4 FtDKO. For O-NALT, organs were pooled from each group. (*p<0.05, ** p<0.01, and *** p<0.001).

We examined the expansion of Ag-specific effector and memory CD8 T cell populations in the lungs of WT and FtDKO mice following LCMV Armstrong infection. At day 8 and day 60 post-infection, we observed significant expansion of viral specific CD8 T cells in organs of FtDKO mice (Figure 2A, B). However, CD8 T cell numbers were significantly reduced (∼50%) in the lungs of FtDKO mice compared to WT mice, consistent with the described role of selectin-mediated homing to inflamed lung (Figure 2C, D). Similar to a recent study, CD8 T cell numbers in the MedLN following infection remained severely reduced (∼85%, Figure 2C, D) [21]. Interestingly, we noted that at day 60, Ag-specific memory CD8 T cells in MedLN and O-NALT were not reduced (Figure 2B, D). In fact, FtDKO LN showed significant enrichment of Ag-specific memory CD8 T cells compared to the total CD8 population (Figure 2 and J. R. Harp and T. M. Onami manuscript in preparation).

### Naïve and memory T cells preferentially accumulate in non-lymphoid organs in FtDKO mice

Paradoxically, our results following infection show that effector and memory Ag-specific CD8 T cell populations were reduced in the inflamed lungs of FtDKO mice, while naïve CD8 populations were increased in the lungs of uninfected FtDKO mice (Figures 1 and 2). Previously published studies have shown that defective T cell migration to inflamed lungs was due to loss of expression of selectin ligands by T cells—i.e. defective migration to inflamed tissues in FtDKO mice is T cell intrinsic [25]. However, defective T cell migration to LNs is due to loss of expression of selectin ligands on HEVs [16], [25]. In FtDKO mice, selectin ligand expression is absent in all cells, including activated T cells.

**Figure 3:**
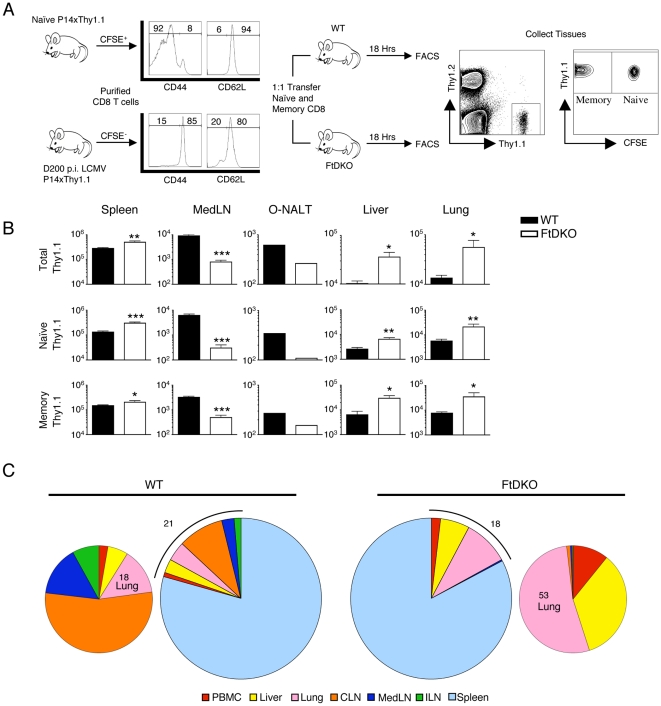
T cells preferentially accumulate in non-lymphoid organs of FtDKO mice. CD8 T cells were purified from naïve Thy1.1xP14 Tg mice (CFSE^+^) or immune (day 200 p.i.) Thy1.1xP14 Tg chimeric mice (CFSE^-^), transferred into WT or FtDKO recipient mice, and recipient mice were sacrificed 18 hours later. Thy1.1 was used to differentiate endogenous from transferred T cell populations from indicated organs. *A*, Experimental design and phenotype of transferred cell populations. *B*, Enumeration of transferred CD8 T cell populations in indicated organs. *C*, Pie charts showing the distribution of Thy1.1 cells in WT and FtDKO recipient mice in spleen, PBMC, liver, lung, CLN, ILN, and MedLN. Of the indicated organs, ∼80% of transferred cells are located in spleen. The smaller pie chart shows the distribution of the remaining transferred cells. Data was collected from n = 5 WT and n = 6 FtDKO from two independent experiments. (*p<0.05, ** p<0.01, and *** p<0.001).

To determine whether naïve or memory CD8 T cells, capable of expressing selectin ligands, selectively homed to the lung of FtDKO mice, we performed adoptive transfer of *bona fide* P14.Tg WT naïve and central memory CD8 T cells into WT or FtDKO recipient mice under non-inflammatory conditions. As expected, transferred T cells were significantly impaired in homing to LNs as well as O-NALT in FtDKO mice (Figure 3B). In contrast, we observed that transferred naïve and memory CD8 T cells show increased numbers in the spleen, lung, and liver of FtDKO mice compared to WT mice (Figure 3B, C). We saw no evidence of transferred T cells in the bronchial alveolar lavage (BAL) (data not shown). The majority of transferred CD8 T cell populations were located in the spleens of both WT and FtDKO mice (79% and 82% respectively). The remaining CD8 T cells show differences in their distribution comparing blood, liver, lung, cervical lymph node (CLN), iliac lymph node (ILN), and MedLN (Figure 3C). In WT recipient mice, the majority of the remaining transferred CD8 T cells preferentially homed to the LNs with a small fraction in non-lymphoid organs (Figure 3C). In contrast, in FtDKO mice, the majority of the remaining transferred CD8 T cells were preferentially located in the lung and liver, with few entering the LNs (Figure 3C). Thus, consistent with our observation of increased numbers of naïve T cells in non-lymphoid organs of FtDKO mice, these data demonstrated that naïve and central memory CD8 T cells preferentially accumulated in non-lymphoid organs in FtDKO mice.

### Inhibition of naïve T cell trafficking to LN by Pertussis toxin treatment results in selective re-distribution to non-lymphoid organs

**Figure 4:**
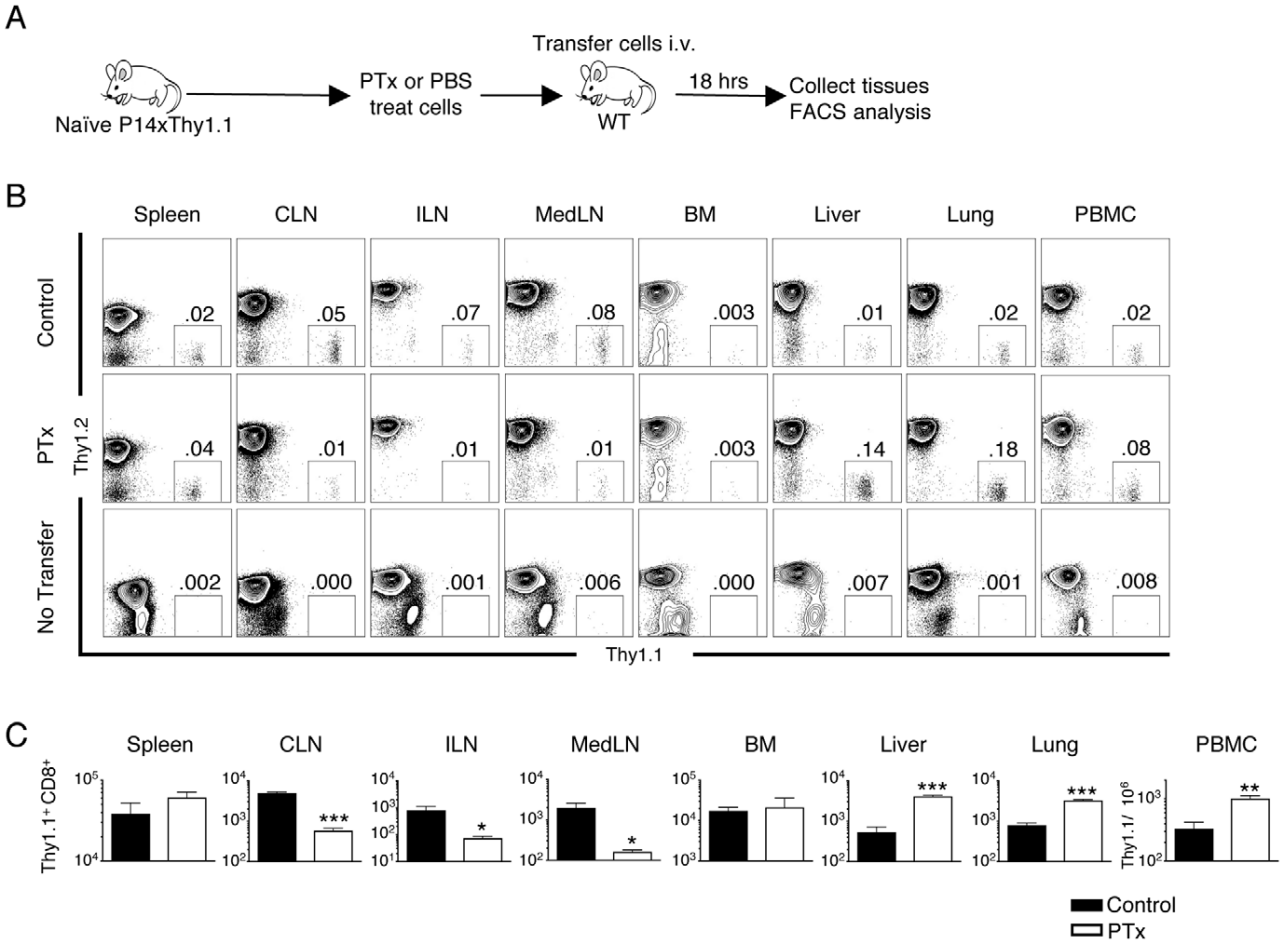
Inhibition of T cell trafficking to LN by pertussis toxin treatment results in selective re-distribution of naïve T cells to non-lymphoid organs. Splenocytes from Thy1.1xP14 Tg mice were treated with either pertussis toxin (Ptx) or PBS control and transferred into WT recipient mice. 18 hours post transfer, T cells were isolated from indicated organs and analyzed by FACS. *A*, Experimental design. *B*, Representative FACS analysis of indicated organs gating on CD8 T cells. Numbers indicate percentage of transferred cells in the indicated organ. FACS panels from animals that received untreated cells, Ptx-treated cells, or received no transferred cells are shown. *C*, Enumeration of transferred cells in organs. Data was analyzed from n = 3 control untreated and n = 4 Ptx treated groups. (*p<0.05, ** p<0.01, and *** p<0.001)

The observation of increased numbers of T cells in the lungs of FtDKO mice could be due to several possibilities. One possibility is that loss of selectin ligand expression in the FtDKO mice results in a change in the lung environment, making the lung more permissive for T cell entry. A recent report observed that reduced sLe^x^ expression on lung epithelial cells contributes to defects in airway wound repair [31]. Additionally, defects in core fucosylation have been associated with abnormal lung development and an emphysema-like phenotype in Fut8 deficient mice [32], [33]. Another possibility is that by blocking T cell entry to the LN, T cells are re-distributed to other sites. Naïve T cells may end up in non-lymphoid organs, if these sites are part of the normal migratory pathway of naïve T cells [34], [35], [36]. To test this hypothesis, we examined whether inhibition of chemokine receptor signaling by pertussis toxin treatment (Ptx) also results in increased migration of naïve CD8 T cells to lung and other non-lymphoid organs of recipient mice. In Figure 4, we show that as expected, Ptx treatment of naïve CD8 T cells resulted in significantly impaired trafficking of transferred T cells to LNs, observed as decreased percentages and overall numbers of transferred Thy1.1 cells in CLN, ILN, and MedLN (Figure 4B, C). In contrast, significantly more naïve CD8 T cells were observed in the lung and liver (Figure 4C). We observed no differences in numbers of transferred naïve CD8 T cells in spleen and bone marrow. Notably, we observed an increased concentration of transferred Ptx treated T cells in the blood (Figure 4C). Additionally, as shown later, if we treated recipient mice with blocking antibodies to L-selectin (anti-CD62L), we also observed reductions in naïve CD8 T cells in LNs, and increased T cells in lung and liver. Taken together, these data support our hypothesis that general inhibition of T cell trafficking to LN results in increased T cell re-distribution to non-lymphoid organs.

### Retention of T cells in LN by FTY720 treatment results in reduced naïve CD8 T cell populations in non-lymphoid organs

**Figure 5:**
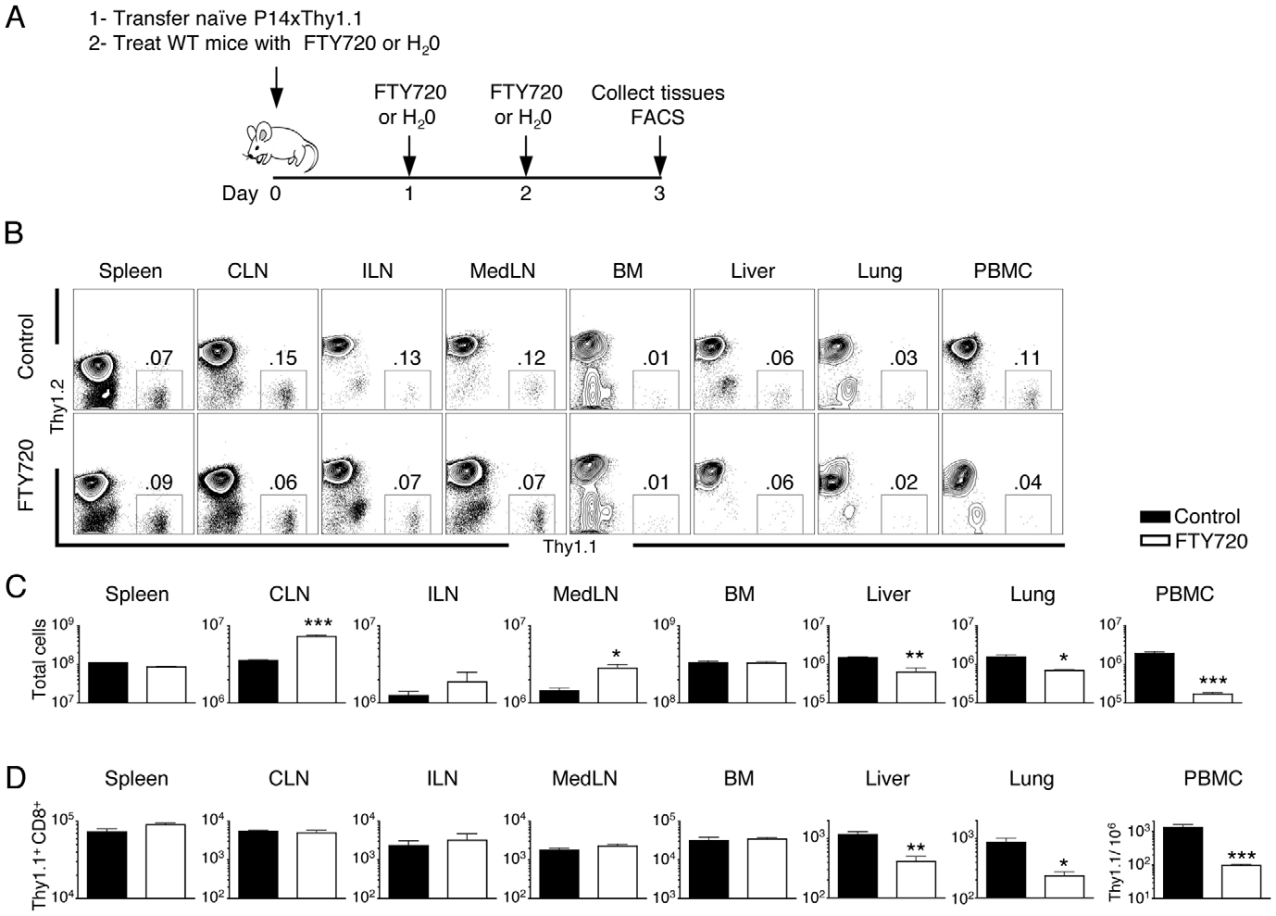
Retention of naïve T cells in LN by FTY720 treatment depletes naïve T cell populations in non-lymphoid organs. Splenocytes from naïve Thy1.1xP14 Tg mice were adoptively transferred into WT recipient. Recipient mice were treated for three days with FTY720 and lymphocytes in organs analyzed by FACS. *A*, Experimental design. *B*, Representative FACS analysis of indicated organs. Gating on CD8 T cells, numbers indicate percentage of transferred cells. *C*, Total lymphocyte numbers isolated from indicated organs. *D*, Transferred Thy1.1^+^ CD8^+^ T cells from indicated organs. Data from one representative experiment is shown, with n = 2 control and n = 3 FTY720 treated mice. (*p<0.05, ** p<0.01, and *** p<0.001).

Thus far, our experiments demonstrated that when we inhibited entry of naïve CD8 T cells via HEVs to LNs, we observed increases in the number of T cells in lung and liver (Figure 3–4). Based on this result, we predicted that if we blocked T cell egress from the LN, we should observe reduced T cells in the lung and liver. To test this hypothesis, we transferred naïve CD8 T cells into recipient mice, and treated these mice with FTY720, an agonist for S1P1, which inhibits T cell egress from the LN [37], [38]. Treatment with FTY720 generally resulted in increased total lymphocytes in LNs and a significant drop in total lymphocytes in the PBMC (Figure 5C). FACS analysis and enumeration of the transferred naïve CD8 T cell population revealed that transferred T cells in treated mice were significantly lower in the liver, lung, and PBMC while remaining unchanged in other organs (Figure 5D). Our study using *bone fide* naïve T cells supports and extends data in a recent paper showing FTY720 treatment depletes endogenous naïve phenotype T cells in peripheral organs [38].

### Relationship of naïve T cells in non-lymphoid organs and T cells in blood

**Figure 6:**
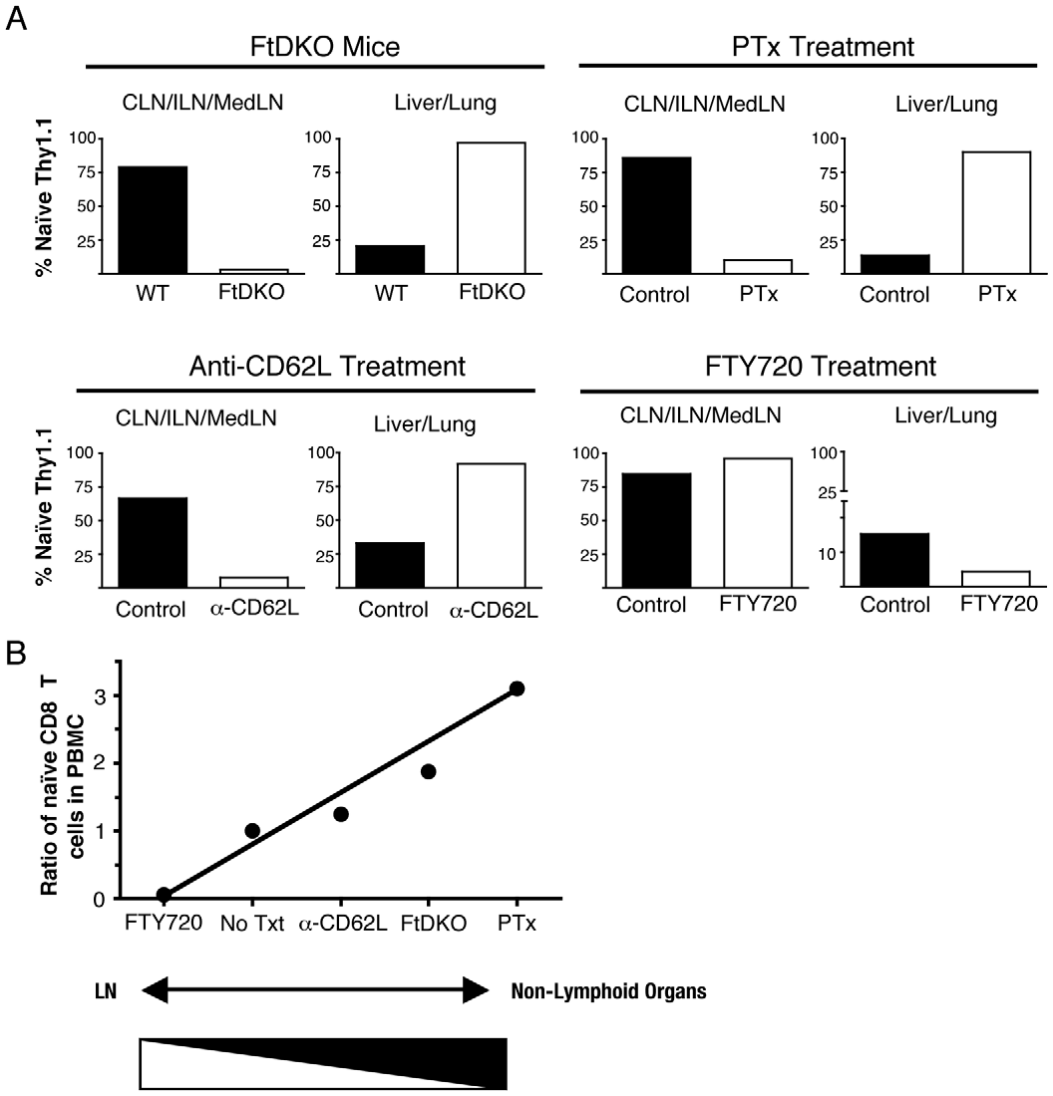
Naïve T cell migration to non-lymphoid organs is influenced by T cell concentration in the blood. Proportions of transferred naïve T cells in selectin ligand deficient mice (FtDKO), Gαi signal disruption (Ptx), CD62L blockade, and FTY720 treated mice are shown. *A*, Total number of transferred naïve CD8 T cells in CLN, MedLN, ILN, liver, and lung was calculated, and the percent of transferred naïve CD8 T cells in LNs versus non-lymphoid organs is shown for each treatment group. *B*, The relationship of naïve CD8 T cells in the blood compared to naïve CD8 T cells in organs (LN versus non-lymphoid organs). Concentration of naïve CD8 T cells in PBMCs was determined for each animal and the ratio of treatment versus control for each treatment group is shown (R = 1 for control, untreated group). Data is shown from 5 independent experiments for a total of n = 28 mice.

If we focused our analysis on naïve CD8 T cells, a clear inverse relationship emerges from our data (Figure 6). We used several methods to block T cell entry to LN, including genetic ablation of selectin ligands (FtDKO mice), inhibition of G-coupled dependent chemokine receptor signaling in T cells, or anti-CD62L treatment of mice to inhibit CD62L dependent migration (Figure 6). In general, under conditions of impaired migration to LN, we observed increased migration to non-lymphoid organs such as lung and liver (Figure 6A). In contrast, S1P1 agonism by FTY720 treatment of mice results in retention of transferred naïve CD8 T cells in LN, but resulted in a drop in naïve T cells in non-lymphoid organs (Figure 6A). Taken together, the proportion of naïve T cells located in non-lymphoid organs was inversely correlated with the proportion of naïve T cells in the LN (Figure 6A).

Under normal conditions naïve T cells emerge from the thymus and migrate via the circulation into secondary lymphoid organs and exit LNs via the lymphatic vessels, culminating at the thoracic duct, which drains into the superior vena cava, returning T cells to the blood. We reasoned that treatments that inhibit naïve T cells in the blood from entering the LN via HEVs would result in higher proportions of naïve T cells remaining in the blood. This higher proportion of T cells in the blood could result in higher numbers of T cells that transit into the lung via the lung's dual blood supplies, the pulmonary and bronchial arteries, where T cells could accumulate in the lung perivascular and interstitial compartments [34], [39]. However, in the mouse it is generally agreed that there is limited blood supply from the bronchial arteries, so the pulmonary arteries are the only blood supply to the lung, and T cells may enter lung compartments after being sequestered in the pulmonary capillary bed [40], [41], [42]. Conversely, treatments that retain T cells in the LN could reduce the proportion of T cells in the blood, limiting the number of T cells that may potentially enter the lung.

We analyzed the proportion of transferred naïve CD8 T cells in the blood following inhibition of LN entry, or inhibition of LN exit. For each treatment, we calculated the ratio of the concentration of naïve T cells in the blood in treated versus untreated groups of mice. We then considered whether this resulted in more T cells in LN or non-lymphoid organs. Schematically, we show that there is an inverse relationship between the concentration of naïve T cells in the blood and localization in LN versus non-lymphoid organs (Figure 6B). Generally, when we inhibited naïve CD8 T cell entry to LN, via the HEVs using CD62L antibody blockade, genetic deficiency of selectin ligands, or inhibition of chemokine receptor signaling via G-coupled receptor signaling pathways, we observed more naïve CD8 T cells in the blood, and we also observed more naïve CD8 T cells in non-lymphoid organs (Figure 6B). Conversely, when we inhibited naïve CD8 T cell exit from the LN, via S1P1 agonism, fewer naïve CD8 T cells were observed in the blood, and we observed fewer naïve CD8 T cells in non-lymphoid organs (Figure 6B). Under homeostatic conditions, the majority of naïve T cells preferentially home from the blood via HEVs into the LN, and we observe a small, but measurable population of naïve T cells in non-lymphoid organs (Figure 1 and 6). Thus, our observation of increased naïve T cells in the lungs of FtDKO mice is consistent with our hypothesis that impaired T cell migration to secondary lymphoid organs such as LN, results in increased T cells in the blood, and subsequently results in migration and preferential accumulation in non-lymphoid organs.

## Discussion

The focal event of the adaptive immune response is the encounter between naïve T cells and APCs displaying specific antigen, which results in T cell activation. Thus, our understanding of the immune system will be critically dependent on characterizing the molecular events that regulate and guide highly migratory T cells and APCs to appropriate microenvironments [43]. In this study, we have discovered a previously unknown phenotype in FtDKO mice. We observed increased numbers of naïve T cells in the lungs with no evidence of iBALT formation, and we demonstrated that T cells selectively re-distribute to the lung, as well as several other organs in FtDKO mice (Figure 1, 3). Viral infection of FtDKO mice to induce inflammation and trigger viral-specific T cell expansion, demonstrated that while there was robust CD8 T cell expansion and viral clearance by day 8, Ag-specific effector and memory CD8 T cell populations were significantly reduced in the lungs of FtDKO mice, consistent with the described role of selectin ligands in T cell extravasation to inflamed organs (Figure 2 and data not shown). Taken together, our data suggests that both selectin ligand-dependent and selectin ligand-independent mechanisms contribute to T cell migration to non-lymphoid organs under inflammatory and non-inflammatory conditions, respectively.

To further understand the mechanism of T cell re-distribution and accumulation in non-lymphoid organs under non-inflammatory conditions, we transferred *bona fide* naïve and memory CD8 T cells and enumerated them in organs. However, it must be considered that T cell migration is a very dynamic process and our analysis was limited to one time point (18hrs post-transfer). Earlier or later times may have shown differences in organ distribution of these transferred T cells. Nonetheless, our results demonstrated preferential accumulation of both naïve and central memory T cell populations in non-lymphoid organs of FtDKO mice (Figure 3). This observation is in contrast to the main dogma that suggests these cells do not enter non-lymphoid organs, but fits well with alternatively proposed migration models that suggest T cells continuously enter lymphoid and non-lymphoid organs [44], [45], [46]. Moreover, we found that conditions that inhibit T cell trafficking to the LN, and increase the proportion of T cells in the blood, such as Ptx treatment and anti-CD62L blockade, also result in increased naïve CD8 T cell trafficking to non-lymphoid organs (Figure 4 and 6). In contrast, inhibition of T cell exit from the LN by FTY720 agonism of S1P1, which drops the proportion of T cells in the blood, significantly reduced the number of naïve CD8 T cells in lung and liver (Figure 5 and 6). Similarly, transfer of naïve CD4 T cells into anti-CD62L treated recipient mice or FTY720 treated mice also showed alterations of CD4 T cell numbers in the lung (S. Caucheteux and W.E. Paul, unpublished observation). Taken together, these data suggest that when T cell trafficking to LN is altered, this influences the concentration of T cells in the blood that enter the spleen and non-lymphoid organs such as the lung and liver. While this does not preclude the possibility that additional changes in peripheral tissues of FtDKO mice may also influence trafficking of T cells, it does suggest that altered T cell trafficking to LNs is sufficient to cause increased T cell accumulation in several organs.

Normal re-circulation of T cells to secondary lymphoid organs such as LN, spleen, and mucosa associated lymphoid tissues such as O-NALT, is thought to be important for T cell interaction with stromal cells expressing IL-7 and other survival cytokines [47], [48]. As noted above, there is controversy in the literature regarding whether naïve T cells normally migrate into non-lymphoid organs. Several reviews and textbook discussions report that only effector and memory T cells enter non-lymphoid organs, and naïve T cells do not normally enter these sites [8], [44], [49]. However, other studies have reported that as part of the normal migratory pathway, naïve T cells enter non-lymphoid organs including lung, but naïve T cell residency time in these organs is short compared to effector/memory T cells [34], [35], [36], [45], [50], [51]. Recently published data has shown that lung and liver are also sites of significant IL-7 production as measured by RNA message level [52]. Possibly, naïve T cells that migrate into highly vascularized organs such as liver and lung are able to receive sufficient IL-7 survival signals that sustain them in these microenvironments. Moreover, several recent papers have shown that T cells are capable of responding to viral antigens in organs and T cells are not dependent on trafficking to draining lymph node for priming [53], [54], [55]. Neo-lymphoid aggregates or follicles have been described in both liver and lung, and are proposed to be sites of potent T cell immune responses [54], [55].

The importance of elucidating mechanisms by which T cells, as well as other inflammatory cells, migrate into and out of the lung is underscored by the global health burden associated with lung diseases including COPD, asthma, and lung metastasis. Our data examining uninfected FtDKO mice revealed substantial increases in naïve T cells in the lung, and uncovered a selectin ligand independent pathway of T cell migration in the absence of lung inflammation. Several years ago, another group published a study describing selectin-independent mechanisms of trafficking to the inflamed lung following *Mycobacterium tuberculosis* infection of FtDKO mice [21]. Similar to our data, they showed that activated CD8 T cell numbers were reduced in the lung early and late following infection, but these mice were able to control the infection comparable to WT mice [21]. However, the study's authors concluded that recruitment of effector T cells to lungs was solely selectin-independent, and argued that the decreased numbers they observed following infection was due to a delay in priming of T cells. Since LCMV efficiently primes T cells in the spleen, where we observed no defects in T cell migration, we conclude that our observation of decreased Ag-specific CD8 T cells in lung was likely due to the contribution of selectin ligands in T cell homing to inflamed lung consistent with earlier work [23], [25], [27]. However, our study is consistent with Schreiber et. al. 's conclusion that substantial numbers of activated CD8 T cells are able to migrate into the lung and peripheral organs in a selectin ligand-independent manner [19], [21].

Selectin mediated binding initiates the earliest step of lymphocyte extravasation by slowing lymphocyte rolling along HEVs. As demonstrated, the loss of selectin ligand expression results in profound impairment in T cell extravasation to LN [16]. However, for T cell entry to the lung parenchyma, it is possible that physical slowing in the small capillaries of the lung is sufficient for T cells to efficiently enter lung routinely as part of normal migration [34], [35], [51]. Several studies have documented that the normal diameter of a capillary in the lung is ∼7 µm which requires that larger leukocytes, such as lymphocytes and granulocytes, undergo cytoskeletal rearrangement for cell deformation and elongation in order to pass through pulmonary capillary beds [40], [56], [57]. Otherwise, these cells become sequestered at these sites. If so, when the proportion of lymphocytes in the blood increases, such as under inflammatory conditions, or when T cell entry to the LN is blocked, T cell entry to the lung may increase as T cells become trapped in the capillary bed, and move into adjacent perivascular and interstitial compartments of the lung. Thus, selectin-mediated binding likely enhances an already effective entry process to the inflamed lung. Furthermore, additional cell adhesion molecules expressed by effector and effector memory T cells also enhance trafficking, as well as retention, in peripheral organs such as lung [58], [59]. The presence of antigen and/or changes in chemokine expression in tissues following microbial infection will also influence naïve T cell migration/retention in non-lymphoid organs [60].

In summary, our data discovered that FtDKO mice had a significant increase in naïve T cells in the lungs, and demonstrated increased naïve and central memory T cell accumulation in non-lymphoid organs. Furthermore, we observe an inverse relationship between naïve T cell location in LN and non-lymphoid organs. By inhibiting T cell entry to or exit from the LN, we show that the ratio of naïve T cells in the blood is altered, and this influences the number of naïve T cells that re-distribute to non-lymphoid organs. In patients with leukocyte adhesion deficiency diseases, where patients suffer from recurrent infections, including a significant proportion of respiratory infections, altered leukocyte trafficking may influence disease development in certain organs [61], [62], [63]. Proposed immunosuppressive therapies that target or alter trafficking of T cells into or out of the LN should consider that such treatments will likely influence the distribution of T cells in blood, and consequently, increase T cell accumulation in non-lymphoid organs, including the lung [64], [65].

## Materials and Methods

### Mice and Immunizations

Fucosyltransferase IV and VII double knockout (FtDKO) mice backcrossed at least 9 generations to C57BL/6 were originally generated by Dr. John Lowe [16]. Mice were obtained from the Consortium for Functional Glycomics (Scripps Research Institute, La Jolla, CA) and bred in-house as homozygous double knockouts, but breed poorly. Transgenic P14xThy1.1 mice were provided by Dr. Rafi Ahmed (Emory Vaccine Center, Atlanta, GA) and bred in-house. C57BL/6 (WT) mice were purchased from The Jackson Laboratory. Memory P14xThy1.1 chimeric mice were generated by adoptive transfer of 5×10^5^ total splenocytes from transgenic P14xThy1.1 mice into C57BL/6 recipients and infected with 2×10^5^ pfu LCMV-Armstrong. FtDKO and C57BL/6 wildtype control mice were immunized intraperitoneally (i.p) with 2×10^5^ pfu LCMV-Armstrong. All mice were maintained under specific pathogen-free conditions at the University of Tennessee in accordance with IACUC guidelines and used at age 2 to 6 months. All animals were handled in strict accordance with good animal practice as defined by University of Tennessee IACUC committee and all animal work was approved by the committee.

### Naïve and Memory Cell Trafficking

Where indicated, MACS (Miltenyi Biotech, Auburn, CA) purified naïve and memory P14xThy1.1 CD8 T cells (2×10^6^) from the spleen were adoptively transferred into WT or FtDKO recipient mice. Naïve cells were labeled with carboxyfluorescein succinimidyl ester (CFSE) in order to differentiate transferred naïve and memory P14xThy1.1 CD8 T cell populations. Equal numbers of Ag-specific CD8 T cells were injected intravenously (i.v.) via tail vein. In pertussis toxin (Ptx) inhibition experiments, P14xThy1.1 splenocytes were pretreated with Ptx (Calbiochem, Gibbstown NJ) or PBS for 90 minutes at 37°C [50] and 1×10^6^ Ptx or PBS treated Ag-specific CD8 T cells were adoptively transferred into WT recipient mice. For anti-CD62L experiments, 100 µg of MEL-14 (α-CD62L) blocking antibody (BioXcell, W. Lebanon, NH) was injected i.v. into WT recipient mice. Four (4) hours post treatment, 1×10^6^ P14xThy1.1 CD8 T cells were injected i.v. into mice. In FTY720 experiments, 1×10^6^ P14xThy1.1 CD8 T cells were adoptively transferred into recipient WT mice, followed by treatment with 1 mg/kg FTY720 (Calbiochem) or water i.p. with treatment every 24 hrs for 3 days total.

### Organ Harvest, Flow Cytometry, and Histology

At indicated time points mice were sacrificed, organs were perfused with 5 ml cold PBS, and tissues harvested for processing as previously described [22], [66]. Briefly, perfused organs were minced, incubated with HBSS+ 1.3 mM EDTA solution for 30 minutes at 37°C, re-suspended in 225 U/ml type I collagenase for 60 minutes at 37°C, and cells were centrifuged using a Percoll density gradient to isolate lymphocytes from the parenchyma. Single cell suspensions were stained with D^b^NP396-404 or D^b^GP33-41 tetramers, provided by the NIH Tetramer Facility (Atlanta, GA), and mAbs (CD8, CD4, CD44, CD62L, Thy1.1, Thy1.2) [67]. Total number of cells in bone marrow was calculated by multiplying the number of cells from two femurs ×7.9 [68], [69]. Numbers of cells in PBMC are shown as per 10^6^ cells. Monoclonal antibodies were purchased from BD (La Jolla, CA). All samples were run on a FACSCalibur (BD Biosciences) and analyzed with FlowJo software (Treestar). For histology, lungs were infused with 1 mL of 50% OCT (Tissue Tek, Torrance, CA) in PBS, embedded in OCT, and frozen on dry ice, and stored at −80°C. Serial sections were cut at 7 µm and stained with hematoxylin and eosin using routine staining procedures. Lung sections were also stained with biotinylated primary Ab, Thy1.2 (1∶1500; BD Biosciences), followed by Alexa Fluor 568 conjugated streptavidin (1∶400; Invitrogen, Eugene, OR), and analyzed using an epifluorescent microscope (Nikon Eclipse E600). For transfer studies, to calculate the proportions of transferred cells in different organs, total Thy1.1^+^ CD8^+^ T cells in indicated organs were enumerated. The number of transferred cells in each tissue was then divided by the total Thy1.1^+^ CD8^+^ cells in examined organs. Statistical significance was determined using unpaired student *t* tests where *indicates p<0.05, ** p<0.01, and *** p<0.001.
